# Early assessment of fluid tolerance (VExUS) and stroke volume (LVOT-VTI) to predict adverse outcomes in emergency department patients with suspected sepsis^☆^

**DOI:** 10.1016/j.aicoj.2026.100143

**Published:** 2026-08-26

**Authors:** Daniel M. Rolston, John P. Forrester, Elizabeth A. Young, Margaret Gorlin, Amanda Dalpiaz, Nicholas Bielawa, Timmy Li, Kate van Loveren, Daniel Jafari, Mathew Nelson, Allison L. Cohen

**Affiliations:** a Northwell, New Hyde Park, New York, United States; b Newark Beth Israel Medical Center, New Jersey, United States; c State University of New York Upstate Medical University, Syracuse, New York, United States

**Keywords:** VExUS, Fluid tolerance, Volume-responsiveness, Fluid-responsiveness, Sepsis, Velocity time integral, Stroke volume

## Abstract

**Background:**

To determine if early Doppler ultrasound assessments of venous congestion with Venous Excess Ultrasound (VExUS) and stroke volume with left ventricular outflow tract velocity time integral (LVOT-VTI) are associated with 24-h adverse outcomes and fluid-responsiveness in Emergency Department (ED) suspected sepsis patients prior to substantial fluid administration.

**Methods:**

A single-center, prospective cohort study of adult ED patients with suspected sepsis recruited between 5/2020 and 12/2023 (exclusions: receipt of >500 mL fluid, intubated, on vasopressors, or existing do not resuscitate/intubate). The primary outcome was a 24-h ordinal measure of mortality, intensive care unit (ICU) admission, or rapid response team (RRT) activation; and the secondary outcome was fluid-responsiveness (increase in LVOT-VTI ≥10% after a 500 mL fluid bolus). Multivariable ordinal and multinomial regression models were used.

**Results:**

545 patients had VExUS and 493 had LVOT-VTI measurements. 24.7% of VExUS 0 versus 56.5% of VExUS 3 patients died/admitted to ICU within 24 h. VExUS scores ≥1 were associated with increased mortality or ICU admission: VExUS 1: OR 2.37 (95%CI: 1.29–4.36); VExUS 2: OR 2.84 (95%CI: 1.64−4.92); VExUS 3: OR 4.09 (95%CI: 2.24−7.45). VTI alone was not associated with increased adverse outcomes. 23.5% of VExUS 0/VTI ≥ 17 cm patients died/admitted to ICU within 24 h versus 57.3% of VExUS 2–3/VTI < 17 cm (OR 3.67 [95%CI: 2.04−6.61]). 63.7% of VExUS 0 versus 21.1% of VExUS 3 were fluid-responsive. VExUS score ≥1 were associated with decreased odds of fluid-responsiveness: VExUS 1: OR 0.35 (95%CI: 0.19−0.63); VExUS 2: OR 0.20 (95%CI: 0.11−0.37); VExUS 3 OR 0.16 (95%CI: 0.07−0.36). 62.1% of LVOT-VTIs 17−20 cm were fluid-responsiveness (OR 2.01; 95%CI: 1.20−3.37) versus 47.3% in the VTI > 20 cm reference group and 45.8% in the VTI < 17 cm group. 62.1% of VExUS 0/VTI ≥ 17 cm patients were fluid-responsive (reference) versus 16.7% of VExUS 2–3/VTI < 17 cm (OR 0.16; 95% CI 0.07−0.34).

**Conclusion:**

In non-ventilated ED patients with suspected sepsis, VExUS scores ≥1 were associated with increased odds of 24-h mortality/ICU admission and RRT activation and decreased odds of fluid-responsiveness. Combining VExUS and LVOT-VTI assessments early in sepsis may improve prediction of adverse outcomes and better characterize fluid tolerance/responsiveness to guide individualized resuscitation, but further research is needed.

## Introduction

### Background

Determining optimal fluid administration in sepsis remains challenging. While the Surviving Sepsis Campaign (SSC) and the Center for Medicare and Medicaid Services recommend 30 mL/kg within three hours for septic shock [[1], [2], [3]], excessive fluid increases mortality and acute lung injury [4,5]. The SSC also suggests dynamic fluid responsiveness monitoring but lacks specific recommendations for ultrasound for venous congestion [3]. Although a recent trial found no mortality difference between restrictive and liberal strategies, participants had already received >2 L of fluid at enrollment [6].

Given patient heterogeneity in sepsis, early personalized fluid administration may improve outcomes [7,8]. Importantly, fluid responsiveness indicates a patient is on the ascending portion of the Frank-Starling curve but does not alone indicate fluid administration will be beneficial, as historical studies targeting supranormal cardiac output demonstrated harm [9,10]. Moreover, fluid responsiveness can coexist with venous congestion [11], underscoring that fluid responsiveness and fluid tolerance are complementary but independent physiological assessments that together may better inform resuscitation decisions.

Doppler ultrasound can assess both fluid-responsiveness and venous congestion. The Venous Excess Ultrasound (VExUS) score, a novel point-of-care ultrasound (POCUS) assessment, grades venous congestion from 0 to 3 (higher scores indicate more congestion) using inferior vena cava (IVC) diameter, and Doppler ultrasound waveform analyses of the hepatic, portal, and renal veins [[12], [13], [14]]. VExUS is associated with acute kidney injury in post-cardiac surgery patients [13], right atrial pressure [15], and echocardiographic measures of cardiac function in the intensive care unit (ICU) [16]. While the IVC is easily measured [17,18] and initial studies demonstrating its dynamic measurements predict of fluid-responsiveness in sepsis [[19], [20], [21]] have been contradicted by subsequent meta-analyses showing lower accuracy [17,22] and a randomized trial finding no benefit to IVC variability-guided fluid management [23]. Recent studies have sought to better understand the relationship between VExUS score, cardiac function, and filling pressures [[24], [25], [26]]. However, there is no data on the use of VExUS early in the evaluation of septic patients in the ED.

Left ventricular outflow tract (LVOT) velocity time integral (VTI) is a common Doppler echocardiographic measurement of stroke volume [27,28]. In shock states, LVOT-VTIs <16 cm suggest cardiogenic, obstructive, or hypovolemic shock, while LVOT-VTIs >20 cm suggest distributive shock or high output heart failure [29]. Additionally, LVOT-VTIs <17 cm are associated with low ejection fraction [30] and higher cardiac death [31]. A 10–15% increase in LVOT-VTI after a fluid challenge or straight leg raise suggests fluid-responsiveness [[32], [33], [34], [35]], including septic shock [36]. One study found it was a better predictor of fluid-responsiveness than IVC collapsibility index in sepsis [37], and a recent meta-analysis demonstrated stroke volume variation, as well as pulse pressure variation and plethysmographic variability index, were superior to central venous pressure or IVC variation for predicting fluid-responsiveness [22].

The primary objective was to assess whether early Doppler ultrasound assessments of venous congestion (VExUS) and initial stroke volume (LVOT-VTI) predict 24-h adverse outcomes including mortality, intensive care unit (ICU) admission, and rapid response team (RRT) activation in non-ventilated ED patients with suspected sepsis prior to substantial fluid administration; and secondarily fluid-responsiveness (defined as an increase in LVOT-VTI ≥10% after a 500 mL fluid bolus).

## Methods

### Study design and setting

We conducted a prospective, partially-blinded, cohort study of a convenience sample of adult patients with suspected sepsis in a single quaternary care ED between May 21, 2020 and December 18, 2023 when trained physicians or physician assistants were available. Patients were managed by the clinical team. The Strengthening the Reporting of Observational Studies in Epidemiology (STROBE) Statement was followed in the design and reporting of this study [38].

### Selection of participants

Trained study personnel identified patients for inclusion. Eligible patients were ≥18 years who were screened through two methods: 1) an overhead “Code Sepsis” announcement, which is made for all ED patients with potential infection and two of the following: a) fever ≥38.3 °C or hypothermia <36 °C; b) hypotension with systolic blood pressure <90 mmHg or mean arterial pressure <65 mmHg; c) heart rate ≥120 beats per minute; d) respiratory rate ≥24 breaths per minute; or 2) an electronic medical record alert is activated for all patients with two of the above criteria at any point during their ED stay, or one of the above criteria and a white blood cell count >12.0 × 10⁹/L or <4.0 × 10⁹/L, lactate >2 mmol/L or band neutrophils >10%. The treating physicians were then approached to determine if the patient had suspected sepsis requiring antibiotics and admission. We excluded patients if physicians had a high-suspicion for COVID-19. We excluded patients who received >500 mL of fluids prior to enrollment; were on vasopressors or already intubated/ventilator-dependent prior to screening; were unable to provide consent or legally authorized representative was not available; had do-not-intubate (DNI), do-not-resuscitate (DNR), or palliative measures only; and pregnant patients. Patients were followed from enrollment in the ED to hospital discharge.

### Interventions

Sonographers performing the Doppler ultrasound examination included five ultrasound fellowship-trained emergency physicians, two ultrasound fellowship-trained physician assistants, two critical care fellowship-trained emergency physicians, and 15 emergency medicine ultrasound fellows. All sonographers participated in a 45-minute didactic training which reviewed the Doppler ultrasound protocol. The principal investigator precepted their first five study scans prior to being approved as an enrolling investigator. Patients were managed and treated by the clinical team. If the enrolling investigator was not part of the clinical team, the clinical team was blinded to the ultrasound exam. Enrolling sonographers not part of the clinical team were blinded to laboratory results and medical history, though it was impossible to blind them to information overheard during the sonogram or patient appearance.

The Doppler ultrasound protocol involved scanning patients in either a supine or semi-recumbent position. A low frequency phased array transducer, using either cardiac or abdominal pre-settings, was used for the ultrasonographic examination. The VExUS examination first assessed the IVC. If the IVC was ≥2 cm, the sonographers assessed the hepatic, portal, and renal veins with pulsed-wave Doppler to obtain the VExUS score, which was previously described [13,14,39]. If the patient had substantial respiratory movement and could comply, they were asked to hold their breath momentarily to obtain Doppler assessments. The sonographer then moved to the apical 5-chamber view and oriented the pulsed-wave Doppler ultrasound at the LVOT parallel to the direction of blood flow. They obtained three LVOT-VTI measurements for sinus and five measurements for atrial fibrillation rhythms and recorded the mean LVOT-VTI. All data were entered into a Research Electronic Data Capture (REDCap) database. This Doppler ultrasound protocol was repeated by the same sonographer after completion of a 500 mL fluid bolus, if fluids were ordered by the treating clinical team.

### Measurements

VExUS stratifies patients into 4 categories. VExUS 0 patients have an IVC that is <2 cm; VExUS 1 have an IVC that is ≥2 cm without severely abnormal venous Doppler blood flow patterns in the hepatic, portal, or renal veins; VExUS 2 have an IVC that is ≥2 cm and 1 vessel with severely abnormal flow patterns; VExUS 3 have an IVC that is ≥2 cm and ≥ 2 vessels with severely abnormal flow patterns [14]. A blinded second reviewer retrospectively reviewed and scored 40 VExUS examinations (10 randomly selected for each VExUS category) on QPathE. Based on the distribution of the data and previous research demonstrating worse outcomes with LVOT-VTI <17 cm [31], LVOT-VTI measurements were categorized into 1) <17 cm), 2) 17−20 cm, 3) >20 cm [29]. LVOT-VTI measurements could not be retrospectively re-measured by a second reviewer.

Patient demographic and medical record data were collected by study personnel who were trained by the principal investigators for chart abstraction and blinded to the ultrasound findings and study objectives. The 24-h outcomes mortality, ICU admission, and RRT activation were followed by the research coordinator and confirmed by a non-enrolling investigator. Eventual source of infection (bacterial, viral, both, unknown, or no source) was determined from both research coordinator and physician chart review. Bacterial infection was characterized as any infection with a likely bacterial source (e.g., bacteremia, lobar pneumonia or non-viral pneumonia, urinary tract infection, abscess, cellulitis, osteomyelitis, intrabdominal infection). Viral infection was characterized as any viral testing positivity or negative viral testing with suspected viral etiology and negative bacterial cultures. Because the medical record data abstraction was completed with a standardized data collection form and none of the other variables abstracted required clinical interpretation, dual data abstraction or inter-rater reliability assessment of chart reviews was not performed.

### Outcomes

The primary outcome was an ordinal outcome including mortality, ICU admission, RRT activation, or none of the above within 24 h of ED arrival. Patients were categorized into their worst outcome subgroup within 24 h; therefore, if a patient had an RRT and was subsequently admitted to the ICU, their recorded 24-h outcome is ICU admission. The secondary outcome was fluid-responsiveness, which we defined as a ≥10% increase in LVOT-VTI after a 500 mL fluid bolus [40].

### Analysis

We did not perform a formal sample size calculation due to limited literature on VExUS at the time of study design, and our lack of pilot data on VExUS scores, LVOT-VTI estimates, 24-h outcomes, or fluid-responsiveness in our septic ED population. Instead, we adopted a pragmatic enrollment strategy. Presuming at least 50% of patients would fall into the VExUS 0 category, we initially aimed to enroll 450 patients, to ensure >50 patients were enrolled in each of the VExUS 1–3 categories. We increased to 550 patients after an interim analysis revealed approximately 10% were missing LVOT-VTI and 20% did not receive any fluid boluses.

Descriptive statistics (medians and interquartile ranges [IQRs] for continuous variables, frequencies and proportions for categorical variables) were determined for the study sample by VExUS score and LVOT-VTI category. Due to quasi separation from low mortality (n = 15, 2.8%) yielding unreliable regression estimates when mortality was modeled separately, we combined mortality and ICU admission into a single category for the VExUS and VExUS/VTI category regression models. We acknowledge that combining mortality and ICU admission into a single category merges outcomes of different clinical severity. The composite outcome is therefore primarily driven by ICU admissions, and readers should interpret accordingly. This reduced the 24-h ordinal outcome to three categories: 1) mortality/ICU admission, 2) RRT, and 3) none.

For the primary analysis, only the LVOT-VTI analysis met the proportional odds assumption for ordinal regression analysis. Partial proportional odds models were developed for the VExUS and VExUS/VTI category analyses to model the association between the three 24-h ordinal response categories and relevant covariates using cumulative logits to quantify patient response at a given or more extreme category. Unlike proportional odds models which require the odds of response at a given or more extreme category to be proportional (“proportional odds assumption”), partial proportional odds (PPO) models allow for non-proportional relationships between the covariates and cumulative logits; however, this further limited our variable selection. We performed univariate analyses for variables associated with adverse outcomes that are available early in the assessment of septic ED patients (initial systolic blood pressure [SBP] (p < 0.01), respiratory rate (p = 0.06), and lactate (p < 0.01); and Charlson Comorbidity Index [CCI] at time of admission, p = 0.01). Heart rate (p = 0.93), oxygen saturation (p = 0.93), body mass index (BMI) (p = 0.93), history of malignancy (p = 0.77) and transplant (p = 0.27), and source of infection (p = 0.98) were not significant predictors of adverse outcomes and were dropped to improve model fit. History of heart failure (p = 0.01), chronic kidney disease (p = 0.05), and age (p = 0.19) are included in the CCI. VExUS and LVOT-VTI groupings were created based on distribution of the patients into each subcategory and evidence demonstrating worse outcomes with VExUS ≥2 [41,42] and LVOT-VTI <17 cm [31]. We performed complete case analysis for the LVOT-VTI and VEXUS/VTI categories, excluding the patients with missing initial LVOT-VTI measurements.

Secondarily, multinomial regression models were used to assess the association between VExUS scores, LVOT-VTI categories, and combined VExUS and LVOT-VTI categories on the three nominal fluid-responsive categories: fluid-responsive, patient not given fluids, and not fluid-responsive. We excluded patients who did not have an initial LVOT-VTI measured as this was need to quantify fluid-responsiveness. We adjusted for variables deemed to be clinically relevant to fluid administration and available to ED physicians early in the management of septic patients (age; initial SBP, respiratory rate, and lactate; history of heart failure [HF] or chronic kidney disease [CKD]). Heart rate, oxygen saturation, BMI, history of malignancy, transplant, and source of infection were also assessed and dropped after univariate analysis. CCI was not a significant predictor of fluid-responsiveness. To improve clinical relevance and avoid small sample sizes in the combined VExUS and LVOT-VTI categories analyses, we combined VExUS 2 and 3 since both groups show evidence of severe congestion, and we dichotomized LVOT-VTI into <17 cm versus ≥17 cm. We created figures illustrating the estimated predicted probability of outcomes from the multivariable ordinal and multinomial regression models for the primary and secondary outcomes. Inter-rater reliability was measured with weighted Cohen’s kappa statistic.

Statistical analyses were performed using SAS (SAS Institute, Cary, North Carolina, USA) and R (R Foundation for Statistical Computing, Vienna, Austria) by a biostatistician who was not involved in study design or data collection.

## Results

### Characteristics of study subjects

**Fig. 1:**
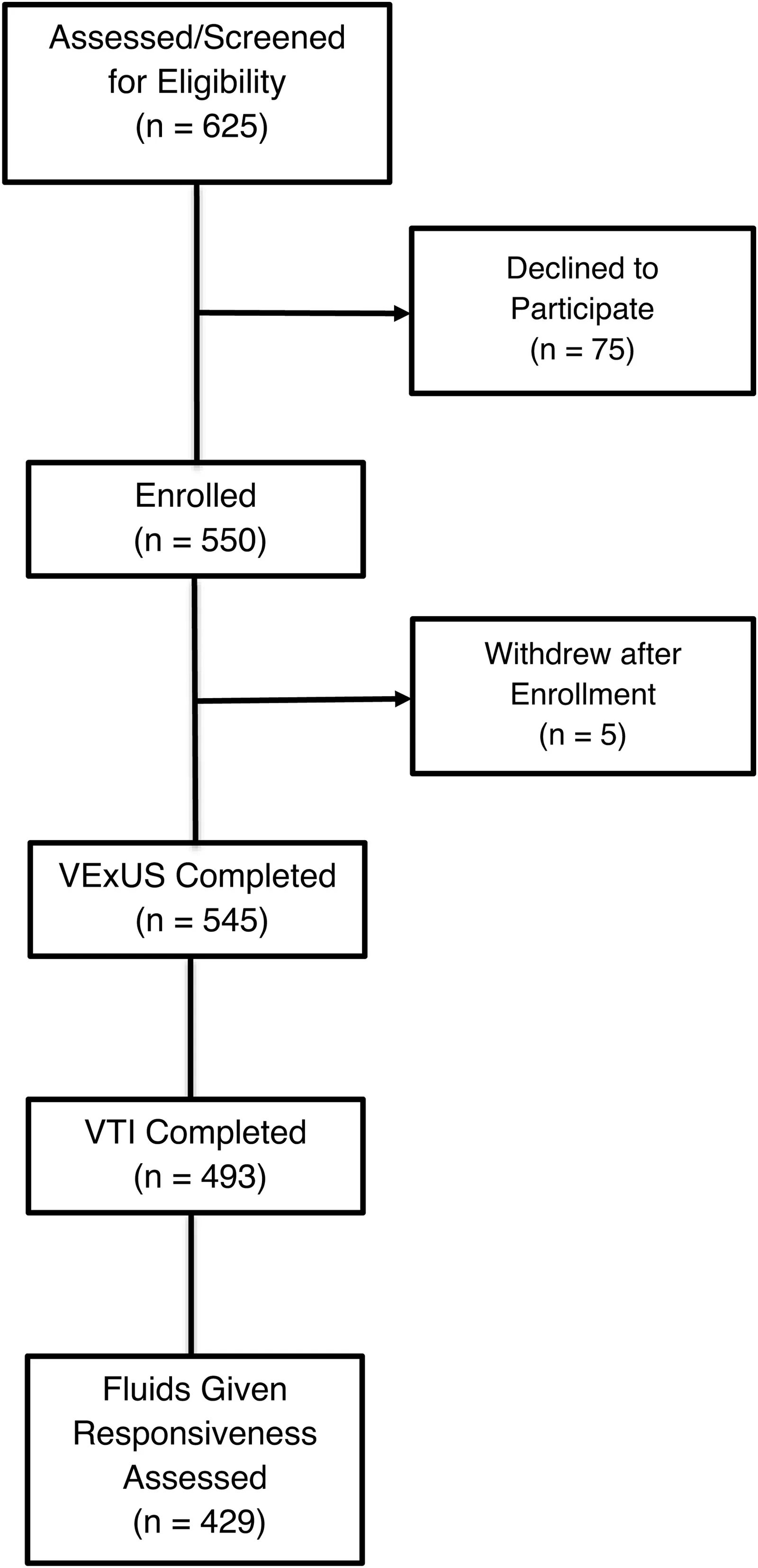
Study flowsheet.

We enrolled 545 patients (Fig. 1). In 256 patients (47%), the treating physician was also a study investigator. Our patient population is elderly, predominantly white, and has a high prevalence of chronic conditions (Table 1). 26.8% had viral sepsis and 12.1% had no source of infection identified. 61.7% had a VExUS of 0, 11.7% a VExUS of 1, 15.2% a VExUS of 2, and 11.4% a VExUS of 3. For those who had an IVC ≥ 2 (VExUS ≥1) triggering venous Doppler assessments, 99% obtained portal vein and 95.7% hepatic vein waveforms, but only 51.7% obtained renal vein waveforms. Patients with higher VExUS scores had higher creatinine and troponin levels, increased prevalence of heart failure and other cardiac diseases, pulmonary hypertension, chronic kidney disease (CKD), and chronic obstructive pulmonary disease, and were less likely to have an identified source of infection.

**Table 1:** Patient demographics and emergency department management by VExUS and VTI.

	VExUS Score 0	VExUS Score 1	VExUS Score 2	VExUS Score 3	Low VTI < 17 cm	Normal VTI 17–20 cm	High VTI > 20 cm
	n = 336 (61.7%)	n = 64 (11.7%)	n = 83 (15.2%)	n = 62 (11.4%)	n = 215 (43.6%)	n = 155 (31.4%)	n = 123 (24.9%)
**Demographics**							
Age, median [IQR]	73 [62, 82]	76.5 [63.5,87.5]	78 [70, 86]	79 [72, 88]	77 [67, 86]	73 [64, 83]	73 [62, 82]
Female Sex	168 (50.0%)	30 (46.9%)	33 (39.8%)	24 (38.7%)	90 (41.9%)	74 (47.7%)	70 (56.9%)
Race							
White	198 (58.9%)	41 (64.1%)	61 (73.5%)	41 (66.1%)	147 (68.4%)	92 (59.4%)	69 (56.1%)
Black	52 (15.5%)	6 (9.4%)	7 (8.4%)	10 (16.1%)	26 (12.1%)	21 (13.6%)	18 (14.6%)
Asian	54 (16.1%)	9 (14.1%)	9 (10.8%)	7 (11.3%)	22 (10.2%)	30 (19.4%)	22 (17.9%)
Other	32 (9.5%)	8 (12.5%)	6 (7.2%)	4 (6.5%)	20 (9.3%)	12 (7.7%)	14 (11.4%)
Ethnicity							
Hispanic	28 (8.3%)	2 (3.1%)	6 (7.2%)	3 (4.8%)	10 (4.7%)	11 (7.1%)	13 (10.6%)
BMI (kg/m^2^)	25.3 [22.3, 29.2]	27.8 [23.1, 32.1]	25.3 [21.9, 30.9]	25.6 [22.3, 30.3]	25.4 [22.1, 30.2]	25.5 [23.0, 29.8]	25.7 [22.5, 30.9]
Weight (kg)	70.4 [59.0, 83.8]	74.8 [61.2, 95.3]	72.0 [61.2, 83.9]	71.7 [59.0, 85.3]	72.6 [59.0, 85.7]	72.6 [61.4, 83.9]	69.4 [57.2, 85.7]
**Vitals**							
Systolic Blood Pressure	109 [92, 132]	119 [98, 141]	116 [91, 140]	108 [89, 132]	107 [89, 129]	118 [96, 135]	119 [93, 142]
Diastolic Blood Pressure	67 [57, 78]	69 [59, 81]	67 [54, 80]	64 [54, 80]	67 [55, 80]	69 [58, 79]	67 [55, 78]
Heart Rate	106 [88, 122]	95 [81, 111]	94 [79, 119]	90 [74, 106]	102 [84, 121]	103 [84, 122]	101 [81, 114]
Respiratory Rate	20 [18,22]	20 [18,22]	20 [18,25]	20 [18,22]	20 [18,24]	20 [18,22]	20 [18,22]
Oxygen Saturation	96.0 [94, 98]	97 [94, 99]	97 [93, 99]	96.5 [94, 99]	96 [94, 98]	97 [94, 98]	96 [94, 98]
**Laboratory Values**							
WBC Count (x 10⁹/L)	11.7 [7.2, 16.3]	11.7 [8.5, 15.6]	11.0 [8.1, 16.3]	11.2 [8.0, 16.2]	11.4 [8.2, 16.7]	11.5 [7.2, 15.0]	12.0 [7.8, 16.7]
Lactate (mmol/L)	2.5 [1.8, 3.5]	2.1 [1.5, 3.4]	2.4 [1.6, 3.7]	2.6 [2.0, 4.0]	2.7 [2.0, 4.4]	2.4 [1.6, 3.4]	2.3 [1.7, 3.1]
Blood pH	7.4 [7.3, 7.4]	7.4 [7.3, 7.4]	7.3 [7.3, 7.4]	7.4 [7.3, 7.4]	7.4 [7.3, 7.4]	7.4 [7.3, 7.4]	7.4 [7.3, 7.4]
Creatinine (mmol/L)	106.1 [79.6, 185.6]	114.9 [88.4, 247.5]	114.9 [79.6, 265.2]	176.8 [123.8, 274.0]	123.8 [88.4, 229.8]	106.1 [79.6, 168.0]	106.1 [79.6, 221.0]
eGFR (mL/min/m^2^)	54 [28, 79]	44 [20, 73]	47 [17, 75]	28 [17,50]	44 [21, 70]	54 [31, 79]	51.5 [21, 77]
High-sensitivity troponin T (ng/L)	41.0 [22.0, 90.0]	64.5 [26.0, 267.5]	55.5 [28.0, 122.0]	65.0 [34.0, 150.0]	69.5 [29.0, 159.0]	42.0 [25.0, 77.0]	37.0 [19.0, 68.5]
**Past Medical History**							
Systolic Heart Failure	49 (14.6%)	15 (23.4%)	24 (28.9%)	36 (58.1%)	78 (36.3%)	20 (12.9%)	10 (8.1%)
Diastolic Heart Failure	13 (3.9%)	10 (15.6%)	11 (13.3%)	18 (29.0%)	28 (13.0%)	14 (9.0%)	7 (5.7%)
Coronary Artery Disease	69 (20.5%)	23 (35.9%)	30 (36.1%)	29 (46.8%)	77 (35.8%)	39 (25.2%)	23 (18.7%)
Atrial Fibrillation	54 (16.1%)	25 (39.1%)	43 (51.8%)	35 (56.5%)	89 (41.4%)	34 (21.9%)	20 (16.3%)
Valvular Disease	26 (7.7%)	15 (23.4%)	14 (16.9%)	23 (37.1%)	38 (17.7%)	21 (13.6%)	8 (6.5%)
Pulmonary Hypertension	5 (1.5%)	2 (3.1%)	3 (3.6%)	7 (11.3%)	10 (4.7%)	2 (1.3%)	1 (0.8%)
Chronic Kidney Disease	44 (13.1%)	10 (15.6%)	17 (20.5%)	20 (32.3%)	41 (19.1%)	21 (13.6%)	16 (13.0%)
End Stage Renal Disease	32 (9.5%)	10 (15.6%)	15 (18.1%)	9 (14.5%)	22 (10.2%)	18 (11.6%)	14 (11.4%)
Malignancy	129 (38.4%)	24 (37.5%)	31 (37.4%)	16 (25.8%)	74 (34.4%)	64 (41.3%)	42 (34.2%)
COPD	31 (9.2%)	13 (20.3%)	22 (26.5%)	12 (19.4%)	34 (15.8%)	21 (13.6%)	10 (8.1%)
Organ Transplant	15 (4.5%)	3 (4.7%)	4 (4.8%)	3 (4.8%)	7 (3.3%)	7 (4.5%)	6 (4.9%)
Type 2 Diabetes	140 (41.7%)	26 (40.6%)	34 (41.0%)	33 (53.2%)	91 (42.3%)	67 (43.2%)	51 (41.5%)
Cirrhosis	12 (3.6%)	2 (3.1%)	3 (3.6%)	2 (3.2%)	3 (1.4%)	5 (3.2%)	6 (4.9%)
Charlson Comorbidity Index	7 [5,9]	8.5 [6,11]	8 [6,11]	8 [7,10]	8 [6,10]	7 [5,9]	7 [5,9]
**Point-of-Care Echocardiography**							
Global Ejection Fraction							
Normal	258 (76.8%)	45 (70.3%)	43 (51.8%)	25 (40.3%)	107 (49.8%)	126 (81.3%)	108 (87.8%)
Hyperdynamic	29 (8.6%)	7 (10.9%)	3 (3.6%)	4 (6.5%)	7 (3.3%)	14 (9.0%)	12 (9.8%)
Moderately Reduced	18 (5.4%)	6 (9.4%)	14 (16.9%)	9 (14.5%)	37 (17.2%)	7 (4.5%)	0 (0.0%)
Severely Reduced	20 (6.0%)	6 (9.4%)	20 (24.1%)	20 (32.3%)	59 (27.4%)	2 (1.3%)	1 (0.8%)
Not Recorded	11 (3.3%)	0 (0.0%)	3 (3.6%)	4 (6.5%)	5 (2.3%)	6 (3.9%)	2 (1.6%)
Right Ventricular Size							
Less than Left Ventricle	217 (64.5%)	23 (35.9%)	10 (12.0%)	6 (9.7%)	74 (34.4%)	85 (54.8%)	80 (65.0%)
Equal to Left Ventricle	88 (26.2%)	27 (42.2%)	45 (54.2%)	25 (40.3%)	87 (40.5%)	44 (28.4%)	32 (26.0%)
Larger than Left Ventricle	20 (6.0%)	13 (20.3%)	27 (32.5%)	29 (46.8%)	53 (24.7%)	21 (13.5%)	8 (6.5%)
Not Recorded	11 (3.3%)	1 (1.6%)	1 (1.2%)	2 (3.2%)	1 (0.5%)	5 (3.2%)	3 (2.4%)
**Source of Infection**							
Bacterial	204 (60.7%)	42 (65.6%)	38 (45.8%)	30 (48.4%)	123 (57.2%)	86 (55.5%)	76 (61.8%)
Viral	82 (24.4%)	19 (29.7%)	31 (37.4%)	14 (22.6%)	61 (28.4%)	41 (26.5%)	26 (21.1%)
Bacterial and Viral	15 (4.5%)	0 (0%)	1 (1.2%)	3 (4.8%)	9 (4.2%)	4 (2.6%)	5 (4.1%)
No Source of Infection	35 (10.4%)	3 (4.7%)	13 (15.7%)	15 (24.2%)	22 (10.2%)	24 (15.5%)	16 (13.0%)
**Treatments in ED**							
Vasopressors	68 (20.2%)	14 (21.9%)	30 (36.1%)	22 (35.5%)	72 (33.5%)	26 (16.8%)	23 (18.7%)
Total Fluid Volume (mL)	2000 [1000,2500]	1000 [500, 1975]	500 [300, 1000]	500 [0, 1000]	1000 [500,2000]	1500 [500, 2200]	1700.0 [1000, 2300]
Total Fluid Volume by Weight (mL/kg)	24.6 [13.7, 33.9]	13.9 [6.2, 23.6]	7.6 [3.7, 14.7]	7.3 [0.0, 11.4]	13.5 [6.3, 26.8]	20.0 [8.9, 32.3]	25.6 [11.7, 32.7]

VExUS = Venous Excess Ultrasound Score, VTI = Velocity Time Integral, BMI = body mass index, WBC = white blood cell, eGFR = estimated glomerular filtration rate.

All 545 enrolled patients had 24-h outcomes recorded. 493 patients (90.5%) had an initial LVOT-VTI measured. LVOT-VTI was >20 cm in 123 (24.9%), 17−20 cm in 155 (31.4%), and <17 cm in 215 (43.6%) (Table 1). Patients with a LVOT-VTI <17 cm had lower initial SBPs, higher lactate and troponin levels, and more heart failure and cardiac disease. Fluid-responsiveness was measured in 429 (78.7%) of enrolled patients after a 500 mL fluid bolus.

### Main results

15 patients (2.8%) died, 164 (30.1%) were admitted to an ICU, and 56 (10.3%) had an RRT at most within 24 h of ED arrival. Of the 429 patients who received fluids, 221 patients (51.5%) were fluid-responsive.

24.7% of VExUS 0 patients died or were admitted to an ICU within 24 h (Table 2). Patients with VExUS scores ≥1 had increased odds of the ordinal outcome 24-h mortality or ICU admission in comparison to a VExUS 0: VExUS 1: OR 2.37 (95% CI 1.29–4.36); VExUS 2: OR 2.84 (95% CI 1.64−4.92); VExUS 3: OR 4.09 (95% CI 2.24−7.45), as well as increased odds of RRT (Table 2, Supplemental S1, S2). A sensitivity analysis adjusting for heart failure and CKD instead of CCI yielded similar results (S3). 63.7% of patients with a VExUS 0 were fluid-responsive and VExUS scores ≥1 were associated with decreased odds of fluid-responsiveness in comparison to a VExUS of 0: VExUS 1: OR 0.36 (95% CI 0.19−0.69); VExUS 2: OR 0.21 (95% CI 0.11−0.41); VExUS 3: OR 0.19 (95% CI 0.08−0.46) (Table 2, S4, S5). VExUS 3 were also associated with increased odds no fluids were given in comparison to VExUS 0 (OR 2.81 [95% CI 1.18−6.73]).

**Table 2:** Outcomes by VExUS score.

	VExUS score			
	0	1	2	3
n (%)	336 (61.7%)	64 (11.7%)	83 (15.2%)	62 (11.4%)
Mortality n (%)	11 (3.3%)	3 (4.7%)	1 (1.2%)	0 (0%)
ICU Admission n (%)	72 (21.4%)	20 (31.3%)	37 (44.6%)	35 (56.5%)
Mortality or ICU^a^ Admission Odds Ratio (95% CI)	Reference	2.37 (1.29–4.46)	2.83 (1.64–4.92)	4.09 (2.24–7.45)
Rapid Response n (%)	20 (5.6%)	9 (14.1%)	15 (18.1%)	12 (19.4%)
Rapid Response^a^ Odds Ratio (95% CI)	Reference	3.17 (1.76–5.72)	4.46 (2.56–7.75)	7.67 (3.80–15.49)
No Fluids Given n (%)	55 (16.4%)	14 (21.9%)	23 (27.7%)	24 (38.7%)
No Fluids Given Odds Ratio^b^ (95% CI)	Reference	1.94 (0.81–4.68)	1.58 (0.70–3.55	2.81 (1.18–6.73)
Fluid-Responsive n (%)	179 (63.7%)	19 (38.0%)	15 (25.0%)	8 (21.1%)
Fluid-Responsiveness Odds Ratio^b^ (95% CI)	Reference	0.35 (0.19–0.63)	0.20 (0.11–0.37)	0.16 (0.07–0.36)

a Multivariable ordinal regression model adjusting for initial systolic blood pressure, initial respiratory rate, lactate, Charlson Comorbidity Index.

b Multivariable multinomial regression analysis adjusting for age, initial systolic blood pressure, initial respiratory rate, initial lactate, history of heart failure, history of chronic kidney disease.

In the LVOT-VTI >20 cm reference group, 2.4% died, 22.8% were admitted to an ICU, and 8.9% had an RRT within 24 h (Table 3). There was no difference in the odds of the ordinal outcome 24-h mortality, ICU admission, or RRT compared with the LVOT-VTI >20 cm reference group (Table 3, S6, S7). 47.3% of patients with a LVOT-VTI >20 cm were fluid-responsive versus 62.1% with a LVOT-VTI 17−20 cm and 45.8% with a LVOT-VTI <17 cm. There were increased odds of fluid-responsiveness in the LVOT-VTI 17−20 cm group compared with the LVOT-VTI >20 cm group (OR 2.01 [95%CI 1.20−3.37]), but no association between the LVOT-VTI <17 cm and >20 cm group (Table 3, Supplemental Figs. S8, S9).

**Table 3:** Outcomes by left ventricular outflow tract velocity time integral.

	LVOT-VTI		
	<17 cm	17–20 cm	>20 cm
n (%)	215 (43.6%)	155 (31.4%)	123 (24.9%)
Mortality n (%)	8 (3.7%)	3 (1.9%)	3 (2.4%)
ICU Admission n (%)	82 (38.1%)	42 (27.1%)	28 (22.8%)
Rapid Response n (%)	26 (12.1%)	13 (8.4%)	11 (8.9%)
24-h Ordinal Outcome Odds Ratio^a^ (95% CI)	1.59 (0.98–2.57)	1.08 (0.65–1.81)	Reference
No Fluids Given n (%)	36 (16.7%)	15 (9.7%)	13 (10.6%)
No Fluids Given Odds Ratio^b^ (95% CI)	1.52 (0.69–3.36)	1.19 (0.50–2.83)	Reference
Fluid-Responsive n (%)	82 (45.8%)	87 (62.1%)	52 (47.3%)
Fluid-Responsive Odds Ratio^b^ (95% CI)	1.30 (0.78–2.18)	2.01 (1.20–3.37)	Reference

LVOT-VTI = Left Ventricular Outflow Tract Velocity Time Integral; OR = Odds Ratio.

a Multivariable ordinal regression analysis for 24-h outcomes mortality, ICU admission, and rapid response activation adjusting for initial systolic blood pressure, initial respiratory rate, lactate, Charlson Comorbidity Index.

b Multivariable multinomial regression analysis adjusting for age, initial systolic blood pressure, initial respiratory rate, initial lactate, history of heart failure, history of chronic kidney disease.

To assess the incremental predictive value of a combined hemodynamic assessment, we compared multivariable models for the primary outcome. A model combining both VExUS and LVOT-VTI categories provided superior statistical fit and discriminative ability compared to models using either VExUS alone or VTI alone (S10). 23.5% of patients in the VExUS 0/LVOT-VTI ≥17 cm group died or were admitted to an ICU within 24 h versus 57.3% in the VExUS 2 or 3/LVOT-VTI <17 cm (OR 3.67 [2.04−6.61]) (Table 4, S11, S12). 62.1% of patients in the reference group with VExUS 0/LVOT-VTI ≥17 cm were fluid-responsive; and the odds of fluid-responsiveness were lower in the VExUS 1/LVOTVTI ≥ 17 (OR 0.30 [95% CI 0.13−0.71]), VExUS 2 or 3/LVOT-VTI ≥17 (OR 0.39 [95% CI 0.18−0.87]), and the VExUS 2 or 3/LVOT-VTI <17 cm group (OR 0.16 [95% CI 0.07−0.34]) (Table 4, S13, S14). Only patients in the VExUS 2 or 3 and VLVOT-VTI ≥17 group had increased odds of not receiving fluids (OR 3.06 [95% CI 1.10−8.47) after adjusting for covariates.

**Table 4:** Outcomes by VExUS and VTI categories.

	VExUS + LVOT-VTI categories					
	VExUS 0 + LVOT-VTI ≥17	VExUS 0 + LVOT-VTI < 17	VExUS 1 + LVOT-VTI ≥17	VExUS 1 + LVOT-VTI <17	VExUS 2/3 + LVOT-VTI ≥17	VExUS 2/3 + LVOT-VTI <17
n (%)	200 (40.6%)	98 (19.9%)	34 (6.9%)	28 (6.4%)	44 (8.9%)	89 (18.1%)
Mortality n (%)	4 (2.0%)	6 (6.1%)	2 (5.9%)	1 (3.6%)	0 (0%)	1 (1.1%)
ICU Admission n (%)	43 (21.5%)	23 (23.5%)	11 (32.3%)	9 (32.1%)	16 (36.4%)	50 (56.2%)
Mortality or ICU Admission OR^a^ (95% CI)	Reference	1.06 (0.59–1.92)	2.78 (1.24–6.24)	1.99 (0.81–4.92)	2.48 (1.16–5.27)	3.67 (2.04–6.61)
Rapid Response n (%)	10 (5.0%)	7 (7.1%)	5 (14.7%)	3 (10.7%)	9 (20.5%)	16 (18.0%)
Rapid Response OR^a^ (95% CI)	Reference	1.15 (0.66–2.01)	3.81 (1.76–8.28)	2.39 (1.00–5.69)	4.61 (2.25–9.50)	6.55 (3.53–12.15)
No Fluids Given n (%)	10 (5.0%)	7 (7.1%)	6 (17.7%)	6 (21.4%)	12 (27.3%)	23 (25.8%)
No Fluids Given Odds Ratio^b^ (95% CI)	Reference	1.77 (0.60–5.21)	1.94 (0.60–6.24)	2.95 (0.86–10.18)	3.06 (1.10–8.47)	2.25 (0.90–4.63)
Fluid-Responsive n (%)	118 (62.1%)	61 (67.0%)	9 (32.1%)	10 (45.5%)	12 (37.5%)	11 (16.7%)
Fluid-Responsive OR^b^ (95% CI)	Reference	1.39 (0.81–2.39)	0.30 (0.13–0.71)	0.57 (0.23–1.42)	0.39 (0.18–0.87)	0.16 (0.07–0.34)

a Multivariable ordinal regression analysis adjusting for initial systolic blood pressure, initial respiratory rate, lactate, Charlson Comorbidity Index.

b Multivariable multinomial regression analysis adjusting for age, initial systolic blood pressure, initial respiratory rate, lactate, history of heart failure, history of chronic kidney disease.

Inter-rater reliability for the overall VExUS score in 40 patients demonstrated good/substantial agreement with weighted Cohen's kappa of 0.72 (95% CI: 0.59−0.85; p < 0.001).

## Discussion

In ED patients with suspected sepsis, VExUS scores ≥1 were associated with increased odds of early adverse outcomes and decreased fluid-responsiveness, after adjusting for covariates. While LVOT-VTI categories show no difference in adverse outcomes, normal LVOT-VTIs (17−20 cm) were associated with increased odds of fluid-responsiveness. Patients with the high venous congestion and low stroke volume had the highest odds of early adverse outcomes and the lowest fluid-responsiveness.

Identifying high-risk septic ED patients is crucial, driving our primary outcome of 24-h mortality, ICU admission, and RRT activations. Notably, the composite outcome of mortality/ICU admission was predominantly driven by ICU admissions (164 vs. 15 deaths), limiting our ability to draw specific conclusions about mortality. RRT was included because, despite our ICU typically admitting only mechanically ventilated or vasopressor-dependent patients, patients receiving other significant interventions (e.g. non-invasive ventilation, diuretics or episodes of hypotension requiring fluid boluses) are also at considerable risk. Early adverse outcomes in sepsis are likely affected by timely interventions like fluid boluses, and delays to ICU admission are harmful [3,43,44]. Our study demonstrates VExUS and LVOT-VTI offer valuable insight into early adverse outcomes, potentially guiding patient disposition.

Studies on the utility of ultrasound for venous congestion have been variable, with recent studies finding conflicting results: one found no association between venous congestion and fluid-responsiveness [11]; the other a weak inverse correlation of VExUS and fluid-responsiveness [45]; and a third finding VExUS worsened with a fluid challenge in fluid non-responders [46]. Our study supports the utility of ultrasound for venous congestion, especially in non-ventilated patients [17,21], by demonstrating VExUS scores ≥1 are associated with increased odds of early adverse outcomes and decreased odds of fluid-responsiveness. Similar to previous research [47,48], patients with venous congestion (higher VExUS scores) also had elevated troponins, increased prevalence of cardiac disease/heart failure, and POCUS findings of decreased contractility and increased RV dilation (Table 1), suggesting patients with elevated VExUS at presentation may reflect underlying cardiac dysfunction rather than acute volume overload alone and represent a high-risk patient population for adverse outcomes. Future prospective research should implement VExUS early in suspected sepsis to determine if it can improve outcomes by identifying etiology of shock or acute illness, thereby guiding disposition and fluid administration. They should also attempt to disentangle whether VExUS serves primarily as a marker of chronic cardio-renal disease severity using early standardized echocardiographic assessments.

VExUS assessments can be time-consuming, particularly for less experienced sonographers in busy environments like the ED. However, only 38% of our subjects, who were often sicker (more chronic illnesses, vasopressor/inotrope use), had an IVC ≥ 2 cm thus triggering the more complex Doppler assessments. The necessity of assessing all three hepatic, portal, and renal veins to quantify venous congestion remains unclear. In our experience, renal vein Doppler waveforms were the hardest to obtain and often were unable to be obtained, potentially leading to under classification of higher VExUS scores. Further research should evaluate the individual predictive ability of these Doppler waveforms to potentially improve VExUS generalizability.

Personalized fluid management for sepsis, balancing fluid-tolerance (venous filling and preload) and fluid-responsiveness (improving blood pressure, cardiac output, and perfusion) may improve sepsis outcomes given the harms of excessive fluid administration [4,[49], [50], [51], [52], [53]]. Unfortunately in EDs, repeat fluid-responsiveness assessments are considered time-consuming and challenging [54], despite evidence that a passive leg raise can be done rapidly [55]. Our goal was to determine if VExUS and/or LVOT-VTI measurements at presentation predict early adverse outcomes and characterize the balance between fluid tolerance and fluid responsiveness. VExUS and fluid-responsiveness assessments together may help clinicians understand where patients fall on the Frank-Starling curve and whether the venous system can accommodate additional volume [56]. Despite minimal fluid administration prior to enrollment, 38% of our suspected septic patients with low venous congestions (VExUS 0) and higher stroke volume (LVOT-VTI ≥17 cm) were not fluid-responsive, questioning fluids indications. Additionally, patients with higher VExUS and LVOT-VTIs <17 cm had high rates of early adverse outcomes (75.2%), suggesting a potential need for closer monitoring and earlier ICU consultation; as well as the lowest fluid-responsiveness (16.7%), demonstrating one out of seven of these patients are fluid-responsive despite not appearing fluid-tolerant. Fluid responsiveness alone is insufficient to guide resuscitation, as increasing cardiac output is not universally beneficial and may cause harm when venous congestion is already present. Nevertheless, these findings highlight the importance of measuring both venous congestion and fluid-responsiveness early for determining how much fluids to administer in suspected sepsis or if fluids are indicated at all. Our pre-planned secondary analysis will evaluate the effects of volume of fluids given on adverse outcomes stratified by VExUS and fluid-responsiveness.

While ED sepsis screening is widespread, 12.1% of patients lacked an identifiable infectious source. These patients had higher VExUS scores, suggesting their chronic heart and renal disease contributed to false-positive sepsis screens. Intriguingly, VExUS 2–3 patients had very low 24-h mortality (1.2% and 0%, respectively) despite high ICU admission, suggesting either their positive sepsis screens were from effective ICU care, decompensated chronic illnesses rather than acute sepsis, or both. Administering fluids without assessing intravascular volume risks misclassifying non-septic etiologies, enabling the negative downstream effects of misdiagnoses [57,58]. Previous studies show ED POCUS can establish diagnoses in undifferentiated shock [[59], [60], [61]]. Early use of VExUS and LVOT-VTI may classify cause of shock (distributive, hypovolemic, cardiogenic, obstructive), enabling tailored treatments to both the septic and non-septic patients who screen positive.

## Limitations

This study has several limitations. First, selection bias in this observational convenience sample may have resulted in sicker patients with higher VExUS scores. We adjusted using multivariable regression, although sample size limited the number of covariates and we did not collect a measure of physician gestalt. Second, the varying sample sizes across analyses could introduce selection bias; however, we assessed the patients who were missing LVOT-VTI for selection bias and found no difference in patient characteristics, VExUS distribution, or outcomes (S15). We also accounted for patients who were not given fluids in our fluid-responsiveness analysis. Third, urgent sepsis care necessitated retrospective inter-rater reliability assessment of VExUS on stored images rather than performing multiple assessments in real time which is much more accurate; therefore, we only performed 40 VExUS reviews for quality assurance and inter-rater reliability testing since our study did not allow for robust inter-rater reliability testing. However, a previous study using three experienced sonographers found high agreement between VExUS scores [39]. Fourth, reliance on highly trained sonographers limits generalizability to less-experienced clinicians. Fifth, the single-center, quaternary-care setting with a chronically ill population limits external validity. Sixth, variable fluid administration speeds may have caused measurement bias. Seventh, using LVOT-VTI as both predictor and outcome risks incorporation bias. Eighth, electrocardiogram gating [62] was not performed during the VExUS assessment because it was not recommended at the time we developed the protocol. Ninth, while global assessments of cardiac function were obtained for most patients (global ejection fraction and right ventricular dilation, Table 1), there was missing data and specific measures of cardiac function (ejection fraction, diastolic dysfunction, right ventricular function) were not routinely measured due to the time constraints of sepsis care. Therefore, they were not included in multivariable models so it is unclear whether these provide similar predictability to VExUS and LVOT-VTI. Tenth, while a Net Reclassification Index (NRI) would be a valuable method for quantifying the incremental predictive value of LVOT-VTI in addition to VExUS, its calculation was not feasible in this study due to the need to re-categorize these measures to meet the sample size requirements of the multivariable models. Instead, we compared model fit and discriminative ability (S10). Finally, sampling bias favored sicker patients, however, this remains the target population for Doppler assessment.

## Conclusion

In summary, in non-ventilated ED patients with suspected sepsis prior to substantial fluid administration, VExUS scores ≥1 are associated with increased early adverse outcomes and decreased fluid responsiveness. LVOT-VTIs of 17−20 cm were associated with increased fluid-responsiveness, but not early adverse outcomes. Combined VExUS and LVOT-VTI assessments may better differentiate 24-h adverse outcomes and fluid-responsiveness, but further research is needed. Future research should evaluate whether early ultrasound-guided assessment of both fluid tolerance (via VExUS) and fluid responsiveness (via LVOT-VTI) can improve individualized resuscitation decisions and clinical outcomes beyond point-of-care echocardiography in suspected sepsis, ideally through a randomized clinical trial.

## Authors' contributions

**Daniel Rolston**: Conceptualization, Methodology, Validation, Formal analysis, Investigation, Writing – Original Draft, Writing - Review, and Editing, Visualization, Supervision, Project administration. **John Forrester:** Formal Analysis, Investigation, Writing – Original Draft, Writing – Review and Editing **Elizabeth Young**: Methodology, Software, Validation, Data Curation, Writing – Review and Editing, Visualization, Supervision, Project administration **Margaret Gorlin**: Methodology, Software, Validation, Data Curation, Formal Analysis, Writing – Review and Editing, Visualization, Investigation. **Amanda Dalpiaz:** Investigation, Writing – Original Draft, Writing – Review and Editing. **Nicholas Bielawa** - Investigation, Writing – Original Draft, Writing – Review and Editing. **Timmy Li**: Conceptualization, Methodology, Formal analysis, Writing – Review and Editing. **Kate van Loveren**: Software, Validation, Data Curation, Writing – Review and Editing. **Daniel Jafari**: Methodology, Investigation, Writing – Review and Editing. **Mathew Nelson**: Conceptualization, Investigation, Writing – Review and Editing. **Allison Cohen**: Conceptualization, Methodology, Validation, Formal analysis, Investigation, Writing – Original Draft, Writing - Review and Editing, Supervision, Project administration.

## Ethics approval and consent to participate

The Northwell Health institutional review board approved this study (Number 20-0151) and written informed consent was obtained from all patients or their legally authorized representatives.

## Consent for publication

All authors give their consent for publication.

## Institutional Review Board

Northwell Health IRB Approval Number 20-0151.

## Declaration of Generative AI and AI-assisted technologies in the writing process

During the preparation of this work the author(s) used **Google Gemini 3 Pro** in order to **edit the text for clarity and word count**. After using this tool/service, the author(s) reviewed and edited the content as needed and take(s) full responsibility for the content of the publication.

## Funding

This study was funded in part by research funding from Flosonics Medical, which was awarded during the course of the study. The funder had no role in the study design, data analysis, or the decision to submit the study for publication.

## Data availability

Upon request.

## Declaration of competing interest

Daniel Rolston receives research funding from the ZOLL Foundation and Flosonics Medical unrelated to the current study. Timmy Li receives research funding from the ZOLL Foundation unrelated to the current. Daniel Jafari receives research funding from the ZOLL Foundation and Theravance Biopharma unrelated to the current study. Allison Cohen receives research funding from Flosonics Medical for a portion of the current study. All other authors have no conflicts of interest to disclose.
