# Supplementary material for: Early assessment of fluid tolerance (VExUS) and stroke volume (LVOT-VTI) to predict adverse outcomes in emergency department patients with suspected sepsis
Source: Ann Intensive Care. 2026 Aug 26;16:100143. doi: 10.1016/j.aicoj.2026.100143 (PMC13564126; doi:10.1016/j.aicoj.2026.100143)
Supplement: Supplementary file 1 [file mmc1.docx]

**Supplemental Figures/Tables**

**S1** Multivariable Ordinal Regression Analysis of 24-hour Outcomes by VExUS

**S2** Probabilities of 24-hour Outcomes by VExUS Score from Multivariable Ordinal Regression Model

**S3** Sensitivity Analysis Controlling for Heart Failure and Chronic Kidney Disease Instead of Charlson Comorbidity Index

|  | **VExUS Score** | | | |
| --- | --- | --- | --- | --- |
|  | **0** | **1** | **2** | **3** |
| **n (%)** | 336 (61.7%) | 64 (11.7%) | 83 (15.2%) | 62 (11.4%) |
| **Mortality**  n (%) | 11 (3.3%) | 3 (4.7%) | 1 (1.2%) | 0 (0%) |
| **ICU Admission**  n (%) | 72 (21.4%) | 20 (31.3%) | 37 (44.6%) | 35 (56.5%) |
| **OR for Mortality or ICU**^a^ **Admission (95% CI)** | Reference | 2.42  (1.31-4.47) | 2.79  (1.61-4.85) | 4.14  (2.16-7.93) |
| **Rapid Response**  n (%) | 20 (5.6%) | 9 (14.1%) | 15 (18.1%) | 12 (19.4%) |
| **OR for Rapid Response**^a^  **(95% CI)** | Reference | 3.17  (1.76-5.72) | 4.46  (2.56-7.75) | 7.67  (3.80-15.49) |

^a^ Multivariable ordinal regression model controlling for initial systolic blood pressure, initial respiratory rate, lactate, history of heart failure, history of chronic kidney disease.

OR = odds ratio,

**S4** Multivariable Multinomial Regression Analysis of Fluid-Responsiveness by VExUS

**S5** Probabilities of Fluid-Responsiveness by VExUS from Multivariable Multinomial Regression Model

**S6** Multivariable Ordinal Regression Analysis of 24-hour Outcomes by VTI Categories

**S7** Probabilities of 24-hour Outcomes by VTI Categories from Multivariable Ordinal Regression Model

**S8** Multivariable Multinomial Regression Analysis of Fluid-Responsiveness by VTI Categories

**S9** Probabilities of Fluid-Responsiveness by VTI Categories from Multivariable Multinomial Regression Model

**S10 Model Fit Statistics Comparing VExUS and VExUS/VTI Categories**

| **Model** | **N** | **AIC** | **BIC (SC)** | **c-statistic (AUC)** |
| --- | --- | --- | --- | --- |
| **VExUS** | 545 | 886.176 | 937.697 | 0.739 |
| **VTI** | 493 | 841.988 | 879.756 | 0.690 |
| **VExUS + LVOT VTI** | 493 | 808.767 | 875.910 | 0.744 |

AIC = Akaike Information Criterion; BIC = Bayesian Information Criterion; c-statistic = Concordance statistic. Lower AIC and BIC values indicate better model fit. Higher c-statistic indicates better discrimination.

**S11** Multivariable Ordinal Regression Analysis of 24-hour Outcomes by VExUS and VTI Groups

**S12** Probabilities of 24-hour Outcomes by VExUS and VTI Groups from Multivariable Ordinal Regression Model

**S13** Multivariable Multinomial Regression Analysis of Fluid-Responsiveness by VExUS and VTI Groups

**Figure S14** Probabilities of Fluid-Responsiveness by VExUS and VTI Groups from Multivariable Multinomial Model

**Table S15**

|  | **LVOT-VTI Present (n=493)** | **LVOT-VTI Missing (n=52)** | **p-value** |
| --- | --- | --- | --- |
| **Demographics** |  |  |  |
| Age, median [IQR] (years) | 75 [64, 84] | 78 [67, 86] | 0.20 |
| Female Sex, n (%) | 224 (45.4) | 27 (51.9) | 0.44 |
| Race, n (%) |  |  | 0.44 |
| White | 321 (65.1) | 37 (71.2) |  |
| Black | 67 (13.6) | 8 (15.4) |  |
| Asian | 84 (17.0) | 4 (7.7) |  |
| Other | 21 (4.3) | 3 (5.8) |  |
| Hispanic, n (%) | 39 (7.9) | 5 (9.6) | 0.81 |
| BMI, median [IQR] (kg/m²) | 25.5 [22.4, 30.2] | 25.3 [22.5, 29.5] | 0.94 |
| **Vitals (Initial)** |  |  |  |
| Systolic Blood Pressure, median [IQR] (mmHg) | 113 [92, 136] | 108 [93, 134] | 0.60 |
| Heart Rate, median [IQR] (bpm) | 100 [83, 118] | 102 [87, 122] | 0.58 |
| Respiratory Rate, median [IQR] (breaths/min) | 20 [18, 23] | 20 [18, 24] | 0.82 |
| Oxygen Saturation, median [IQR] (%) | 96 [94, 98] | 97 [94, 98] | 0.67 |
| **Laboratory Values (Initial)** |  |  |  |
| Lactate, median [IQR] (mmol/L) | 2.5 [1.8, 3.7] | 2.5 [1.8, 3.9] | 0.85 |
| Creatinine, median [IQR] (mg/dL) | 1.2 [0.9, 2.1] | 1.3 [0.9, 2.5] | 0.38 |
| eGFR, median [IQR] | 48 [24, 76] | 43 [19, 63] | 0.20 |
| High-sensitivity Troponin T, median [IQR] (ng/L) | 49 [24, 118] | 42 [22, 97] | 0.43 |
| **Comorbidities** |  |  |  |
| Systolic Heart Failure, n (%) | 114 (23.1) | 12 (23.1) | >0.99 |
| Diastolic Heart Failure, n (%) | 49 (9.9) | 5 (9.6) | >0.99 |
| Coronary Artery Disease, n (%) | 143 (29.0) | 15 (28.8) | >0.99 |
| Atrial Fibrillation, n (%) | 147 (29.8) | 18 (34.6) | 0.53 |
| Valvular Disease, n (%) | 74 (15.0) | 7 (13.5) | 0.84 |
| Pulmonary Hypertension, n (%) | 17 (3.4) | 1 (1.9) | >0.99 |
| Chronic Kidney Disease, n (%) | 86 (17.4) | 9 (17.3) | >0.99 |
| Malignancy, n (%) | 188 (38.1) | 18 (34.6) | 0.69 |
| COPD, n (%) | 72 (14.6) | 8 (15.4) | >0.99 |
| Charlson Comorbidity Index, median [IQR] | 7 [5, 10] | 8 [6, 10] | 0.24 |
| **VExUS Score** |  |  | 0.93 |
| 0 | 305 (61.9) | 31 (59.6) |  |
| 1 | 58 (11.8) | 6 (11.5) |  |
| 2 | 74 (15.0) | 9 (17.3) |  |
| 3 | 56 (11.4) | 6 (11.5) |  |
| **24-hour Outcomes** |  |  |  |
| Mortality or ICU Admission, n (%) | 165 (33.5) | 14 (26.9) | 0.38 |
| Rapid Response Team Activation, n (%) | 50 (10.1) | 6 (11.5) | 0.83 |

Table comparing characteristics of the patients with missing LVOT-VTI versus those with LVOT-VTI measurements. No significant differences were identified to suggest selection bias.
